# One-year outcomes following primary stenting of infrapopliteal steno-occlusive arterial disease using a non-polymer sirolimus-eluting stent: Results from a prospective single-centre cohort study

**DOI:** 10.3389/fsurg.2022.955211

**Published:** 2022-10-05

**Authors:** Konstantinos Tigkiropoulos, Ioannis Lazaridis, Spyridon Nikas, Manolis Abatzis-Papadopoulos, Katerina Sidiropoulou, Kyriakos Stavridis, Dimitrios Karamanos, Athanasios Saratzis, Nikolaos Saratzis

**Affiliations:** ^1^ 1st Surgical Department, Faculty of Health Sciences, Aristotle University, Papageorgiou General Hospital, Thessaloniki, Greece; ^2^ Faculty of Health Sciences, Department of Cardiovascular Sciences, University Hospital Leicester, Leicester, United Kingdom

**Keywords:** chronic limb ischaemia, sirolimus, Cre8, stent, infrapopliteal angioplasty

## Abstract

**Background:**

Clinical outcomes using new generation drug-eluting stents designed specifically for infrapopliteal disease are not widely available, especially in comparison to paclitaxel-based therapies. This series reports 1-year outcomes in patients with diabetes and chronic limb threatening ischaemia (CLTI) undergoing angioplasty, with a sirolimus-eluting tibial stent (Cre8, Alvimedica, Turkey), evaluating the feasibility, safety, and efficacy of this new device. Outcomes were compared to matched patients undergoing infrapopliteal angioplasty using a paclitaxel-coated balloon (DCB).

**Patients and Methods:**

Patients with diabetes and CLTI requiring infrapopliteal intervention were recruited prospectively to undergo angioplasty and primary stenting using the Cre8 sirolimus-eluting stent between January 2018 and October 2020 at a single high-volume vascular centre; outcomes were compared to a group of patients with diabetes and CLTI who had undergone infrapopliteal angioplasty using a DCB. All patients were followed up for at least 12 months using a uniform protocol with duplex ultrasound and examination. The primary outcome measure was target lesion patency (<50% restenosis). Clinically driven target lesion revascularisation (CD-TLR), amputations, Rutherford stage, and mortality were also recorded.

**Results:**

A total of 54 patients (61 target lesions; median age: 69 years, 74% male) were included [27 with the Cre8 device (main group) vs. 27 with a DCB (historical controls)]. Primary patency at 12 months was 81% in the Cre8 group vs. 71% in the control group (*p* = 0.498). Overall, four (15%) patients in the Cre8 group vs. three (11%) patients in the control group underwent a major amputation within 12 months (*p* = 1.0). CD-TLR (all endovascular) did not differ between groups at 12 months (4% Cre8 vs. 10% control group, *p* = 0.599). Rutherford stage improvement at 12 months was superior for the Cre8 group (52% vs. 15% improved by at least one stage, *p* = 0.039). One-year mortality was 15% in the Cre8 group vs. 22% in the control group, *p* = 0.726.

**Conclusions:**

Primary stenting with the Cre8 stent is feasible and safe in diabetic patients and CLTI. When compared to patients undergoing angioplasty with a DCB, there were no significant differences regarding primary patency, CD-TLR, major amputations, and mortality at 12 months. Those treated with a Cre8 stent were more likely to have an improvement in their Rutherford stage.

## Introduction

Peripheral arterial occlusive disease (PAOD), in the form of intermittent claudication or chronic limb threatening ischaemia (CLTI), is an important health problem with increasing prevalence (1). Moreover, CLTI is a limb and life-threatening pathology that requires urgent revascularisation in order to prevent amputation or mortality (2). Patients with CLTI are typically known to suffer from a number of comorbidities, with diabetes being very prevalent among them (2–5). Further, patients with CLTI typically have a steno-occlusive disease of their infrapopliteal arteries, with tibial calcified atherosclerotic plaques being very common (2). Multilevel steno-occlusive arterial disease is also common frequent those with CLTI (2).

Percutaneous transluminal angioplasty (PTA) is a common treatment in the context of PAOD, recommended, for certain patients, by international guidelines (2, 6). The durability of PTA in the infrapopliteal segment is, however, challenged by issues such as vessel diameter, lesion length, presence of calcified atherosclerotic plaques or multilevel disease, and neointimal hyperplasia. Drug-coated balloons (DCBs) and drug-eluting tents (DESs) are increasingly used to address hyperplasia in this clinical context (PAOD), to improve, potentially, patency, and clinical outcomes (7). Randomised studies suggest the superiority of paclitaxel-based endovascular therapies in other segments (e.g., femoro-popliteal arteries) over plain balloon angioplasty (PBA) or bare metal stenting in terms of vessel patency (7, 8). At the same time, some studies have suggested a potential relationship between the use of paclitaxel-based endovascular therapies and mortality or even major lower limb amputation in patients with PAOD who undergo endovascular intervention (7, 9).

In recent years, manufacturers have developed angioplasty balloons or peripheral vascular stents which have incorporated alternative agents to paclitaxel, such as everolimus or sirolimus (10–14). Similar to paclitaxel, these agents are meant to reduce intimal hyperplasia and therefore prevent restenosis/occlusion (12–15), which might be beneficial in terms of limb salvage and/or ulcer healing; however, this remains to be proven. Such devices are now widely available for treating infrapopliteal steno-occlusive arterial disease. Previous small-scale randomised controlled trials have confirmed that infrapopliteal everolimus- or sirolimus-eluting stents are superior, in terms of patency, compared to PBA or bare metal stenting. Some evidence suggests improved patency when using drug-based technologies within the infrapopliteal segment when compared with PBA alone in challenging calcified lesions (such as those seen in patients with diabetes, CLTI, and infrapopliteal arterial disease) (16). The existing industry-funded randomised studies assessing the use of limus-eluting infrapopliteal stents have compared the performance of these devices mostly to either PBA or bail-out bare metal stenting. Further, these studies did not take lesion characteristics into account and were underpowered in terms of detecting differences in clinical outcomes such as amputation-free survival.

The Cre8 stent (Alvimedica, Turkey) is a dedicated infrapopliteal sirolimus-coated stent that was widely introduced in the market a few years ago. There is no high-quality prospective study reporting on clinical outcomes in CLTI, especially in the context of diabetes, using this device.

This prospective cohort study aimed to assess the feasibility and safety of using a newly available dedicated infrapopliteal limus-eluting stent in patients with diabetes and CLTI who were treated for infrapopliteal steno-occlusive disease using primary stenting of the target lesion (Cre8 stent). Outcomes were also compared (at 12 months) to matched groups of patients undergoing infrapopliteal angioplasty for CLTI using a paclitaxel-coated balloon, using a uniform follow-up protocol that included regular duplex ultrasound scans.

## Materials and methods

### Study design

This is a prospective single-centre cohort study, recruiting patients from a tertiary referral vascular centre (Papageorgiou General Hospital, Thessaloniki, Greece). Ethical approval was granted in September 2018 by the Papageorgiou General Hospital Research Committee (no reference available). The study was not funded externally. All patients provided written informed consent before taking part. The study was carried out according to the Declaration of Helsinki.

There were two groups of patients included in the study. The main group of patients (recruited prospectively following written informed consent) consists of patients with a diagnosis of diabetes and CLTI who presented between January 2018 and October 2020 and who underwent angioplasty and primary stenting of at least one infrapopliteal atherosclerotic lesion using the Cre8 sirolimus-eluting stent; these were consecutive patients who presented to our unit during that period of time (with CLTI) and who consented to take part in the study and have the Cre8 stent deployed after plain angioplasty (primary stenting). A group of controls consisting of patients with a history of diabetes who had presented previously to the same unit with CLTI and had undergone infrapopliteal angioplasty with a paclitaxel-coated balloon between December 2017 and January 2020 were selected (historical controls). These patients were individually case-matched for age, sex, Rutherford stage, history of end-stage renal disease, and target lesion (infrapopliteal artery) occlusion, with the patients in the main group (Cre8 stent treatment group). There was at least one patent run-off vessel in both groups pre-operatively. There were no further exclusion criteria; all patients with diabetes and CLTI (presence of tissue loss or rest pain) were eligible to take part (real-world study) in the study, with no lesion-related or procedural exclusion criteria. A total of 156 infrapopliteal percutaneous endovascular procedures for CLTI were performed between December 2017 and October 2020 in this centre.

### Definitions and reporting

All definitions and reporting relating to comorbidities, cardiovascular events, and clinical outcomes were based on the Society for Vascular Surgery reporting guidelines (standards) for patients undergoing endovascular procedures for PAOD (16). Reporting was performed as per the STROBE checklist/statement (Appendix 1).

### Procedures

All procedures were performed percutaneously *via* ipsilateral common femoral artery puncture (ultrasound guided) in an operating theatre under local anaesthetic using fluoroscopic control; the main operator was a consultant surgeon in all cases. Systemic heparinisation was used in all cases (75 units/kg) after access was gained and a sheath was inserted in the femoral artery. All patients were given clopidogrel 75 mg pre-operatively and dual antiplatelet therapy (Aspirin and Clopidogrel 75 mg each) for at least 6 months. Patients were started on high-dose statin therapy pre-operatively (if not already taking statin therapy), and that was continued life-long post-operatively.

### Study devices

All patients in the main group were treated with primary stenting using the Cre8 infrapopliteal stent (Alvimedica, Turkey), which is a polymer-free balloon expandable drug-eluting stent manufactured using chromium-cobalt with 70/80 µm strut thickness and a homogeneous metallic frame. The stent is covered by a thin carbon film (0.3 µm). The stent anti-proliferative agent, sirolimus, is loaded in reservoirs on the stent surface and is not incorporated into a polymer, as is the case with other similar devices. The agent is formulated with free fatty acids which act as a carrier, by, in principle, enhancing drug permeability and bioavailability onto the endothelium. All patients in the control group had an infrapopliteal plain balloon angioplasty, followed by angioplasty of the same lesion using a Lutonix 0.14 paclitaxel-coated balloon (Bard Medical, MN, United States). Paclitaxel is homogenously distributed along the length of the Lutonix balloon with a drug dose density of 2 µg/mm^2^, using two excipients, sorbitol, and polysorbate.

### Data collection and follow-up procedures

Information regarding medical history, operation details, baseline parameters of interest (including intra-procedural imaging), and follow-up examinations were all collected in a purpose-built electronic database prospectively by two investigators (KT and NS), who were responsible for all follow-up procedures. Patients were followed up for a minimum of 12 months with three monthly duplex ultrasound assessments of their lower limb arteries as well as a full clinical examination at each appointment. In case of suspicion of target lesion stenosis on the follow-up duplex ultrasonography exceeding 50%, all patients underwent a digital subtraction angiogram. If the >50% stenosis was confirmed, patients underwent angioplasty (plain) of the re-stenosed lesion, with bare metal stenting (left to the operator's discretion) in the case of flow-limiting dissection or residual stenosis exceeding 50%.

### Outcome measures of interest

The main outcome measure of interest was primary patency defined as the absence of reduction in the diameter of the target lesion arterial lumen >50% or peak systolic velocity ratio >2.5 based on duplex ultrasound, in the absence of target lesion revascularisation at 12 months after the date of the index procedure. Secondary endpoints included all and clinically (rest pain or new tissue loss) driven CD-TLR rates, improvement of Rutherford scale, major lower limb amputation (above the ankle joint), and mortality at 12 months.

### Statistical analysis

Statistical analysis was performed using the R package (Version 3.6.3). Continuous variables are presented as mean and standard deviation or median and interquartile range (IQR) depending on their normality assessment, which was performed based on skewness, kurtosis, and the Kolmogorov–Smirnov test. Mann–Whitney *U* test, Chi-square test, and Fischer's exact test were used to compare outcomes as necessary, based on the normality of distributions. Discrete variables are presented as absolute values and proportions. The survival and amputation rates were estimated using the Kaplan–Meier method. The level of statistical significance is set at *p* = 0.05.

## Results

**Figure 1:**
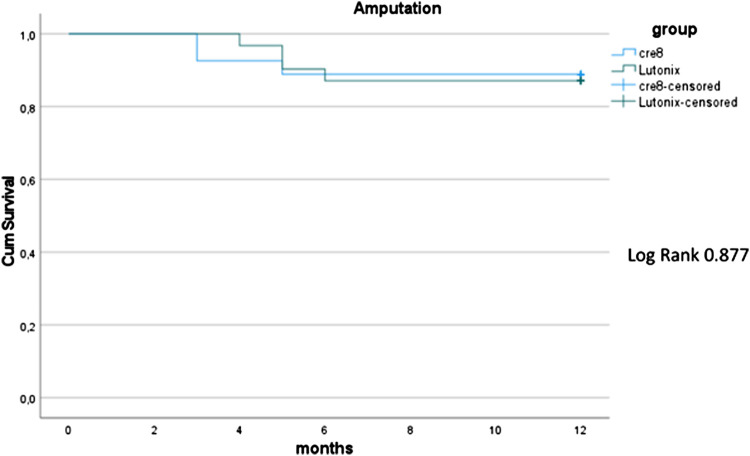
Amputation rate of 2 groups using Kaplan–Meier method.

**Figure 2:**
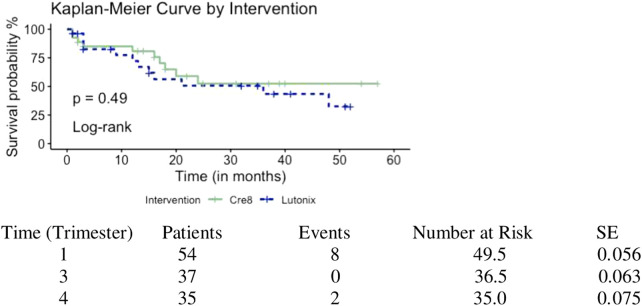
Survival rate of 2 groups using Kaplan-Meier method.

In total, 54 patients (61 target lesions; median age: 69 years, interquartile range: 4 years; 74% male) were included. Twenty-seven patients (main group) were recruited prospectively in the main study group (primary stenting with the Cre8 stent). They were consecutive patients who consented to take part in the study and have the Cre8 stent deployed after plain angioplasty (primary stenting). These 27 patients (main group) were then individually case-matched to a group of 27 patients who had undergone infrapopliteal plain old balloon angioplasty (POBA) followed by paclitaxel-coated balloon angioplasty between December 2017 and January 2020 (with no stents). The demographics and clinical characteristics of the two case-matched groups are listed in Table 1. Most patients were classed as Rutherford category 5 on presentation. All patients in both groups were classified as TASC B. Median lesion length was 20 mm (range 15–40 mm, IQR: 22 mm) in the main study group and 80 mm (range 40–200 mm) in the control group (*p* = 0.001). Fifteen patients were treated for an occlusive infrapopliteal target lesion in each group (55%). The procedures were completed successfully in all cases (100% technical success) in both groups. Synchronous angioplasty of the superficial femoral artery or popliteal artery was performed in 61% of patients (*n* = 16) in the main group and 78% of patients (*n* = 21) in the control group (plain balloon angioplasty in all cases). The tibioperoneal trunk was the most common artery treated in both groups (29% main group and 39% control group) (Table 2). The lesion was cross intra-luminally in all cases in both groups. No low-flow phenomena or distal emboli were observed in either group of patients. Wi-Fi classification scores were not available/recorded as part of this study. All patients in both groups had identical wound care management by the same team of clinicians within the same setting. Those providing wound care were naive to the type of treatment received during the procedure. There were no immediate or 30-day deaths, major cardiovascular events, or access-related complications. No patients were lost to follow-up. Ten patients died within 12 months, four in the main group (15%) vs. six in the control group (22%) (*p* = 0.726). Myocardial ischaemia was the cause of death in six cases, haemorrhagic stroke in one case, and septic shock in three cases. Primary patency at 12 months was 81% for Cre8 treated arteries and 71% for DCB treated target lesions (*p* = 0.498). Major amputation rate at 12 months was 15% (4/27) in the main group vs. 11% (3/27) in the control group, *p* = 1.0 (Figure 1). Regarding Rutherford category scale differences at 12 months, patients in the main group had a significant improvement of their Rutherford scale compared to the control group (*p* = 0.039). Clinically driven target lesion revascularisation rate at 12 months was 4% in the main group (1/23) vs. 9.5% in the control group (2/21) [OR: 2.31, 95% confidence interval: 0.19–27.58, *p* = 0.599] (Table 3). One patient in the main group underwent POBA due to stenosis distal to the stent and two patients in the control group presented with stenosis in the proximal part of previously treated arteries successfully managed with POBA too. Survival between the two groups was not significantly different (Figure 2).

**Table 1:** Demographics and clinical characteristics of patients in the two study groups.

Parameters of interest	Main group—Cre8 stent 27 patients	Control group—Lutonix 27 patients	*p*-value
Age (years)	69 (IQR:4)	69 (IQR: 4)	N/A (case-matched)
Gender (male)	20 (74%)	20 (74%)	N/A (case-matched)
Hypertension	27 (100%)	27 (100%)	—
End-stage renal disease requiring dialysis	3 (11%)	1/27 (4%)	0.610
CAD	9/27 (33%)	13/27 (48%)	0.406
AF	2/27 (7%)	4/27 (15%)	0.699
COPD	1/27 (4%)	3/27 (11%)	0.610
Hyperlipidaemia	3/27 (11%)	4/27 (15%)	1
Previous infrapopliteal angioplasty *plain angioplasty in all cases*	1/27 (4%)	3/27 (11%)	0.610
Previous femoro-popliteal angioplasty *plain angioplasty in all cases*	2/27 (7%)	4/27 (15%)	0.699
Lesion length (mm)	20 (15–40)	80 (40–200)	<0.001

CAD, coronary artery disease; AF, atrial fibrillation; COPD, chronic obstructive pulmonary disorder; IQR, interquartile range.

**Table 2:** Target lesion characteristics per group.

	Main group—Cre8 stent 27 patients; 31 target lesions	Control group 27 patients; 31 target lesions	*p*-value
Tibioperoneal trunk	9 (29%)	12 (39%)	0.429
Anterior tibial artery	8 (26%)	8 (26%)	1
Posterior tibial artery	7 (23%)	4 (13%)	0.327
Peroneal artery	7 (23%)	6 (19%)	0.760
Occlusion	15 (48%)	15 (48%)	1
Non-occlusive stenosis (>50%)	16 (52%)	16 (52%)	1
Superficial femoral artery occlusion	2 (6%)	1 (3%)	0.561
Superficial femoral artery stenosis >50%	8 (26%)	12 (39%)	0.285
Popliteal artery stenosis >50%	12 (44%)	13 (48%)	0.8
Popliteal artery occlusion	1 (3%)	5 (16%)	0.088
Previous angioplasty of target lesion	0	0	—
Severely calcified target lesion (based on duplex imaging at baseline)	17	15	0.618

**Table 3:** Outcomes of interest per group over a 12-month follow-up.

	Main group—Cre8 stent 27 patients; 31 target lesions	Control group 27 patients; 31 target lesions	*p*-value
Primary patency	22/27 (81%)	16/21 (71%)	0.498
Re-intervention	1 (4%)	2 (9.5%)	0.599
Major lower limb amputation (above ankle joint)	4 (15%)	3 (11%)	1.0
Death (all cause)	4 (15%)	6 (22%)	0.726

Clinical outcomes (death, amputation) expressed as proportion of the overall n of patients treated per group. Patency and re-intervention for target lesion stenosis or occlusion expressed as proportion of the overall number of lesions treated per group.

## Discussion

This case-matched series (non-randomised) with prospective uniform follow-up procedures have confirmed the feasibility and safety of infrapopliteal endovascular treatment in patients with steno-occlusive PAOD using primary stenting with a dedicated tibial sirolimus-eluting stent. There were no intra-operative or immediate post-operative adverse events. In a non-randomised comparison with historical controls individually and carefully, case-matched for important clinical parameters known to be associated with adverse outcomes in this group of patients (17, 18), the use of the Cre8 stent showed similar performance to infrapopliteal angioplasty with an established paclitaxel-coated balloon with proven clinical efficacy in treating PAOD below the inguinal ligament as well as specifically in infrapopliteal arteries (19, 20). Those treated with the Cre8 stent primarily were more likely to experience an improvement in their Rutherford stage at 12 months in this series. Patency, limb loss, and mortality rates were similar and not inferior to previously reported clinical and imaging outcomes for patients with diabetes and advanced PAOD in the form of CLTI.

Most previous randomised and observational studies, all of which were funded by industry partners, including patients with infrapopliteal PAOD undergoing endovascular treatments, have compared patients who had drug-eluting as a bail-out or primary procedure vs. PBA or bare metal stenting (typically bail-out stenting) (11, 15). The only randomised study comparing the use of drug-eluting stents (using everolimus, zotarolimus, or sirolimus) vs. drug-coated balloon angioplasty in complex (long) infrapopliteal lesions was the IDEAS trial (18). The primary endpoint was restenosis >50% of the target lesion assessed angiographically at 6 months and the secondary endpoints included immediate stenosis/occlusion after the procedure and the need for revascularisation of the target lesion within 6 months. The study was not powered to detect amputation-free survival or other hard clinical outcomes/endpoints. Long-term outcomes (beyond 6 months) have not been reported either. Patients who underwent angioplasty with a limus-eluting stent (primary stent placement arm) had significantly lower post-procedure restenosis rates (9.6% for the stenting arm vs. 24.8% in the paclitaxel angioplasty arm, *p* < 0.0001), a lower restenosis rate at 6 months (28% vs. 57.9%, *p* = 0.0457), but no significant difference in terms of the need for target lesion revascularisation (7.7% vs. 13.6%, *p* = 0.65). Besides the IDEAS randomised trial, industry-funded trials as a whole have shown a benefit in terms of patency when using drug-eluting stents for complex infrapopliteal lesions vs. PBA or bare metal stents; however, the situation remains unclear with regard to the performance of drug-eluting stents vs. drug-coated angioplasty or other modalities (11, 12, 14, 15). This is an important gap in the literature, especially since drug-coated balloon angioplasty might fare better compared to PBA in complex lesions, both in the femoro-popliteal and infrapopliteal segments (20–22). The control groups, therefore, used in the currently available infrapopliteal drug-eluting stenting randomised trials might not be optimal. Further, these trials have included a fairly small number of patients with diverse lesions and clinical characteristics at baseline. As a result, despite randomisation, their results might not reflect comparisons between homogeneous groups of patients, especially with regard to lesion complexity. Another point of criticism for these trials has been the fact that they employ a number of imaging-based and clinical exclusion criteria, i.e., they might not reflect real-world practice (all-comers), where multilevel disease and major tissue loss are very prevalent.

The overall safety and efficacy of paclitaxel-coated balloon angioplasty of infrapopliteal arteries were evaluated in a systematic review and meta-analysis of all the currently available industry randomised trials by Katsanos et al. (9). The primary endpoint was freedom from all death and major amputation (amputation-free survival) at 12 months. Eight randomised trials with 1,420 patients were included in the analysis. The results indicate significantly inferior survival for the paclitaxel group compared vs. PBA, with a 13.7% risk of death or major amputation for those having paclitaxel-based therapies vs. 9.4% for those who did not receive paclitaxel (hazard ratio 1.52; 95% confidence interval: 1.12–2.07, *p* = 0.008). At the same time, target lesion revascularisation was significantly lower in the paclitaxel arms (11.8% vs. 25.6%, risk ratio 0.53; 95% confidence interval: 035–081, *p* = 0.004). The studies used in the meta-analysis, as discussed above, do suffer from a number of limitations, most importantly the fact that they were not designed or powered to assess clinically driven outcomes (e.g., amputation or survival) and did not randomise all-comers. The outcomes of this recent Katsanos meta-analysis are therefore being strongly debated at the moment.

The Cre8 stent has recently been made available in the wider market for the treatment of patients with infrapopliteal steno-occlusive PAOD. Compared to previous limus-based stents, the Cre8 stent does not use a polymer to bind the immunoproliferative drug (sirolimus) on its surface. The efficacy of the Cre8 DES against other polymer-based drug-eluting stents has been studied in randomised clinical trials in patients undergoing percutaneous coronary interventions; however, there are no data specifically for PAOD. The Reservoir clinical trial (23) was a multicentre, randomised, noninferiority trial that showed acceptable results for the Cre8 stent vs. established polymer-based drug-eluting coronary stents at 9 months, using computed tomographic coronary angiography to assess coronary lesions.

The purpose of this current study was to explore the feasibility and safety profile of the Cre8 stent in infrapopliteal disease treatment in an all-comers prospective registry. We also aimed to compare the efficacy of endovascular treatment with the Cre8 stent at 12 months against a group of homogeneous carefully matched controls, undergoing drug-coated balloon angioplasty, a treatment known to be associated with superior patency rates vs. PBA alone. The Cre8 device in this series fared well in terms of patency against the drug-coated angioplasty controls at 12 months and was not associated with major intra- or post-operative events. Major amputation rates and re-interventions were not statistically different between the two groups.

### Limitations

The main limitations of this study are the lack of randomisation and the single-centre nature of the series; however, the study was primarily designed as a prospective cohort/series in order to assess the feasibility and safety of treatment using this new device. Further, given the small number of patients included in the series and lack of randomisation, we opted to compare against a carefully case-matched (nested individual case matching) group of patients undergoing angioplasty with a paclitaxel-coated device. Due to the number of patients available in the study, introducing lesion length as well as the number of patents run-off vessels as an additional case matching criterion would limit even further the number of patients available for analysis which would increase the chance of type 2 error and selection bias. Further, no industry or other financial support was given, i.e., this is an independent series of all-comers, reflecting real-world practice, without imposing strict selection criteria. Another potential limitation is that vessel lumen patency was evaluated by duplex ultrasound, which is an operator-dependent examination; however, all operators were experienced sonographers and patients underwent a subtraction angiogram in case of concern.

## Conclusion

Treating infrapopliteal arterial lesions in patients with CLTI and diabetes with the newly available Cre8 sirolimus-eluting stent is feasible and safe with results similar to or superior to plain angioplasty followed by the application of drug-coated (paclitaxel) balloons even though the level of evidence is low. Major amputation rate, CD-TLR, and mortality were similar in both groups. More randomised and large observational clinical studies are necessary to establish long-term efficacy/effectiveness and clinical outcomes using the Cre8 stent and the newly available infrapopliteal limus-based endovascular therapies.

## Data Availability

The original contributions presented in the study are included in the article/Supplementary Material, further inquiries can be directed to the corresponding author.
